# Dental Care Utilization among Veterans by Smoking Status

**DOI:** 10.1155/2019/3419805

**Published:** 2019-02-07

**Authors:** R. Constance Wiener, Ruchi Bhandari, Alcinda K. Trickett Shockey, Christopher Waters

**Affiliations:** ^1^Associate Professor, West Virginia University, Department of Dental Practice and Rural Health, School of Dentistry, 104a Health Sciences Addition, PO Box 9415, Morgantown, WV 26506, USA; ^2^West Virginia University, Department of Epidemiology, School of Public Health, Robert C Byrd Health Sciences Center North, Room G104C, Morgantown, WV 26506, USA; ^3^Associate Professor, West Virginia University, Department of Dental Hygiene, School of Dentistry, Robert C Byrd Health Sciences Center North, Room 1192A, Morgantown, WV 26506, USA; ^4^West Virginia University, Department of Dental Research, School of Dentistry, 106a Health Sciences Addition, PO Box 9448, Morgantown, WV 26506, USA

## Abstract

**Background:**

Given the critical importance of dental care utilization among veterans and the overall health consequences of tobacco use in all populations, the purpose of this research is to examine smoking as a risk factor for poor dental care utilization among United States Veterans.

**Methods:**

A secondary data analysis of cross-sectional data from the National Survey of Veterans was conducted. The primary outcome was dental care utilization (Yes, No). Frequency, chi-square analyses, and multivariate logistic regression statistical tests were performed while adjusting for confounding factors.

**Results:**

There were 6,308 veterans in the study. Veterans who were current smokers were less likely to have dental care utilization within the previous six months than former smokers or never smokers. In unadjusted logistic regression analysis, current smokers had an odds ratio of 2.83 [95% CI: 2.36, 3.40] as compared with never smokers. The adjusted odds ratio for current smoking on dental care utilization was 1.71 [95% CI: 1.40, 2.09] as compared with never smoking.

**Conclusions:**

Since veterans who smoked are less likely to have dental care utilization within the previous six months, they are at higher risk for later diagnosis of dental problems. Veterans who smoke should be specifically targeted with interventions to ensure frequent dental visits, so future problems may be averted or managed early in their development.

## 1. Introduction

Keeping veterans healthy is a priority for the Veterans Health Administration. Smoking is a known risk factor for poor health. In the past, the military had a history and culture supporting smoking. Many veterans began smoking during their service. Although 57% of the U.S. population reports *never smoking*, only 32% of veterans are never smokers [1, 2]. However, the percentage of veterans and nonveterans who are *current* smokers is similar (approximately 20%) [2].

There were 19.9 million veterans in the United States in 2017 [1]. Of these veterans, there were 14.8 million under age 65 years, 2.9 million aged 65–69 years, and 2.2 million who were aged 70–74 years; 46.2% were members of the Army (9.2 million) and 81.9% were White (16.3 million) [1]. A total of 6.07 million veterans (31.1%) received care from the Veterans Health Administration (VHA) in 2017 [1]. Veteran healthcare expenditures are categorized in priorities from 1 to 8 with several subsections within each priority. The average healthcare expediture in 2014 varied from $1,111 in priority 8 (subsection G) to $11,702 in priority 1, whereas the average healthcare expenditure for nonveterans was $1,068 [1].

A modifiable behavior, such as smoking, is a risk factor for chronic conditions including metabolic disorders, cardiovascular diseases, and *poor oral health* [3]. In a systematic review, researchers found a dose-response relationship between smoking and tooth loss [4]. Smoking was identified as an independent risk factor for the development and severity of periodontal disease and tooth loss by other researchers [5–7]. Most periodontal disease cases in the U.S. are associated with smoking [8, 9]. Smoking is implicated as a component cause in over three-quarters of oral cancers [10], significantly increasing the risk of oral cancer among smokers [11, 12].

Periodic, regular, and professional dental visits help dentists to prevent, control, delay, or treat dental problems [13]. In a longitudinal study of over 5,000 participants, associations between preventive dental care service utilization and *fewer tooth losses* were observed [14]. Participants who reported smoking or diabetes had lower frequency of tooth loss with biannual dental visits compared with participants who smoked and only had annual visits. Additionally, epidemiologists suggested a link between less dental care service utilization and advanced stage of oral cancer at diagnosis [15, 16]. The main nationally representative data sources for dental care utilization are the Medical Expenditure Panel Survey (MEPS), the National Health and Nutrition Examination Survey (NHANES), and the National Health Interview Survey (NHIS). While estimates of dental care utilization differ among these data sources [17, 18], they remain useful, especially for national trends. Adult dental utilization had been declining from 2003 to 2012 due to a decline in the number of adults with private dental benefits; however, it was stabilized in 2013–2014 [19]. The stabilization may be the result of provisions of the Affordable Care Act in which Medicaid eligibility was expanded and included dental benefits to adults in several states [19].

Within national data sets, there is a lack of information on U.S. veterans. A U.S. veteran is any person who served on active duty in the U.S. Armed Forces (Army, Navy, Air Force, Marine Corps, and Coast Guard). The researchers of existent studies have consistently identified dental care as a critical need for veterans. For example, researchers in a Massachusetts county identified oral health as the greatest unmet need among veterans [20]. Researchers conducting a veterans' needs assessment in California showed dental care among their top three needs [21]. Researchers in a Connecticut county also identified dental care as a significant healthcare concern among veterans [22]. In a study conducted using data from the Veterans Health Administration, researchers concluded that half had periodontitis [13]. Additionally, there are metropolitan/rural differences with rural veterans being less likely to utilize dental services and more likely to have poorer oral health outcomes [23].

Dental emergencies have a negative effect on military operations, should someone be recalled to duty [24]. The Department of Defense identified regular dental visits as critical to individual medical readiness. Given the critical importance of dental care utilization among veterans and the overall health consequences of tobacco use among veterans, the purpose of this population-based analysis is to examine smoking as a risk factor for poor dental care utilization among United States veterans.

### 1.1. Theoretical Framework

The Andersen Expanded Behavioral Model is a theoretical model which is used to explain general healthcare utilization and the risk factors that influence healthcare utilization. It was chosen as the theoretical framework for this study on *dental care* utilization. Andersen proposed categories of risk factors which influence healthcare utilization. These are predisposing factors which are generally immutable factors (such as sex, age, and race/ethnicity), enabling factors (such as marital status, education, insurance, and income), need factors (such as health status), and personal health/behavioral practices (such as *smoking*, drinking, physical activity, and obesity) [25]. Based upon the healthcare utilization model, dental utilization is hypothesized to have similar factors. Smoking, as a personal health/behavioral practice, is being hypothesized as a major contributor to poor dental utilization and fewer dental visits. The Andersen model was used to select the other explanatory or potentially confounding variables in the study (predisposing factors such as sex, age, and race/ethnicity were included; enabling factors such as income, marital status, education, and insurance were included; and the need factor of self-reported health was included). It should be noted that due to (1) data availability, (2) the specific questions posed in the data source, and (3) degrees of freedom, all explanatory or potentially confounding variables could not be included in the final analysis. The rationale for this study is that previous dental utilization studies concerning smoking focused on other groups. For example, U.S. youths in grades 9–12 were studied with results showing an association with smoking and dental visits in the previous 12 months with an adjusted odds ratio of 0.75 [26]. Also, a study of children aged 10–17 years living in a household with smokers less likely to have a dental visit within the previous year was reported [27]. It is important to determine if a similar pattern exists for veterans [28].

### 1.2. Statistical Methodology

The analyses included frequency analyses and weighted percentage estimates of the sample. Bivariate analyses (chi-square analyses) were conducted to determine associations between smoking status, sex, race/ethnicity, age, marital status, education, income, insurance, and self-reported health status with dental care utilization. The level of significance selected, *a priori,* was 0.05. Unadjusted logistic regression analysis and adjusted logistic regression analyses were conducted on dental care utilization by smoking status. The adjusted logistic regression modeling was conducted controlling for sex, race/ethnicity, age, marital status, education, income, insurance, health self-perception, and oral health perception. Due to the number of participants not reporting their type of address and the difficulty in interpreting rurality from the manner in which the question was posed, the type of address was not included in the logistic regression analyses. Due to small sample size of veterans who were females and the small sample size of veterans who were not White, two sensitivity analyses were conducted excluding race as a covariate in one of the analyses and sex as a covariate in the other. All statistical analyses incorporated the weightings and accounted for the complex design of NSV2010. Analyses were conducted with SAS 9.3 (Carey, NC, USA). All independent variables were entered in a single step in the logistic regression model. Variables were selected and included in the model based on their importance to the dental visits shown in the literature.

## 2. Methods

### 2.1. Study Design and Data Source

This study received an acknowledgment as nonhuman subject research (protocol 1511920072) from the researchers' academic institutional review board. A cross-sectional study design was used involving secondary data analyses of previously collected public data from the National Survey of Veterans, 2010 (NSV2010) [28].

The primary aim of the NSV2010 data source was to survey veterans with the purpose of planning and allocating resources for veterans [28]. The NSV2010 was the sixth, and most recent, comprehensive nationwide survey for the Department of Veteran Affairs [28]. The NSV2010 researchers used a cross-sectional research design for their study of noninstitutionalized veterans [28]. They used two sampling approaches: an address-based sampling (ABS) and a list-based sampling approach [28]. Potential address-based participants received a notification, a screening letter, and, if eligible, were sampled for either the mailed or web-based survey. Potential list-based participants received personal contact letters [28]. There were 10,972 surveys mailed and 8,710 surveys returned (66.7% response rate). The surveys were weighted for probability of selection/nonresponse [28]. Data were poststratified to known population totals to represent the noninstitutional veteran population [28].

### 2.2. Study Sample

This study included veterans, aged 20 years and above, from the NSV2010 study who had complete data on the dental visit, smoking status, sex, race, age, marital status, income, insurance adequacy, and self-reported health status. The final study sample included the data from 6,308 veterans.

### 2.3. Measures

#### 2.3.1. Key Dependent Variable

The key variable was dental care utilization within the previous six months (Yes, No). Information for this variable was gathered from the NSV2010 question “In the last six months, have you had any dental care or visited a dentist?” [28]. This was the only dental utilization question posed on the NSV2010 questionnaire.

#### 2.3.2. Key Independent Variable

The key independent variable was smoking status. Participants were asked in the NSV2010 if they had smoked at least 100 cigarettes in their lifetime. The question had dichotomous response categories of “Yes” (to indicate current or former smokers) or “No” (to indicate never smokers). A follow-up question was posed to respondents who endorsed “Yes” to the previous question. The veteran was asked if he or she smoked cigarettes now with “Yes” being used to identify a current smoker and “No” being used to identify a former smoker. Both questions were used to create the smoking variable (current smoker, past smoker, and nonsmoker) for this current study.

#### 2.3.3. Other Variables

Other variables are known to be associated with access and utilization of dental services and/or be potential explanatory or confounding variables. Of these potential variables, the following were available to be included in the analyses: sex (categorized as male or female); race/ethnicity (categorized as White or Other due to the small number of minorities in the survey); age in years (50 years and above, 35 years to less than 50 years , and 20 years to less than 35 years as the 2010 survey did not have participants who were born before 1991; that is, it did not include participants ages 18 years to less than 20 years); marital status (categorized as married or not married); education (categorized as high school graduate or less, some college, college degree or more); income (categorized as less than $20,000, $20,000 to less than $30,000, or $30,000 and above); insurance (categorized as adequate, or inadequate: based on the question “my family has a health insurance plan that adequately covers me and my family” [25] in which the responses “completely agree, agree, and neither agree nor disagree” were categorized as adequate and “disagree and completely disagree” were categorized as inadequate); health self-perception (categorized as excellent/very good/good or fair/poor based on the question, “In general, would you say your health is ... excellent; very good; good; fair; poor?”); oral health perception (categorized as excellent/very good/good, or fair/poor: based on the question, “How would you rate the health of your teeth and gums? Would you say it is excellent; very good; good; fair; poor?”); and mailing address (categorized as rural route, street, U.S.PO box/other box, or missing based on the question, “At which of the following types of addresses does your household receive mail? A street address with a house or building number, an address with a rural route number, a U.S. Post Office box, a commercial mailbox establishment)” [25].

## 3. Results

There were 6,308 veterans in this study sample. Most were male (91.6%) and White (86.9%). Almost two-thirds (64.5%) were current or past smokers and 35.4% reported never smoking. There were 55.3% who reported having a dental visit within the previous six months. Details are presented in Table 1.

The Rao-Scott chi-square test results for dental visits within the previous six months and smoking and other variables are presented in Table 2. Veterans who were current smokers were less likely to have dental care utilization within the previous six months than former or never smokers. Veterans who reported a dental care utilization in the previous six months were more likely to be white, older (50 years of age or older), married, have a college degree or higher, have an annual income of $30,000 or higher, and have adequate insurance and were more likely to report excellent/very good/good general and oral health perception. The unadjusted odds ratio (OR) and adjusted odds ratio (AOR) with their confidence intervals (CIs) of smoking on no report of a dental care utilization within the previous six months are presented in Table 3. In unadjusted logistic regression analysis, current smokers were more likely to not have had dental care utilization as nonsmokers (OR 2.81; 95% CI: 2.34, 3.38; *P* < 0.0001). After controlling for other risk factors and potential confounders, the odds ratio of a current smoker not having dental care utilization in the previous six months as compared with nonsmokers remained statistically significant (AOR 1.50; 95% CI: 1.22, 1.84; *P* < 0.0001). The OR and AOR for the comparison of former smokers with never smokers failed to reach significance. Veterans who did not have dental care utilization within the previous six months were more likely to be other than white (AOR 1.37; 95% CI: 1.08, 1.74); not married as compared with married (AOR 1.24; 95% CI: 1.06, 1.47); educated through high school or less as compared with being educated to college or more (AOR 2.41; 95% CI: 2.05, 2.83); and to have a family income less than $20,000 as compared with $30,000 or above (AOR 2.69; 95% CI: 2.14, 3.38). Other results are presented in Table 3. Three sensitivity analyses to determine covariate effects were conducted. The results were similar to the results presented in Table 3. In the first sensitivity analysis, race/ethnicity was not included among the covariates. In the second analysis, sex was not included among the covariates. In the third sensitivity analysis, self-reported health was not included among the covariates. There were significant associations between smoking categories and education categories in subgroup analysis where the reference categories were never smoking and college degree or beyond. The only exception was that the association between former smoker and college degree or beyond failed to reach statistical significance.

## 4. Discussion

In a large nationally representative sample of U.S. veterans, we found that veterans who were current smokers were more likely not to have dental care utilization within the previous six months as compared with never smoking veterans [AOR, 1.50; 95% CI: 1.22,1.84]. Only 37.0% of veterans who were current smokers reported having dental care utilization within the previous six months compared with 62.3% of never smoking veterans who reported having a dental care utilization within the previous six months (*P* < 0.0001). These results are consistent with the Andersen Expanded Behavioral Model for overall healthcare utilization that explains healthcare utilization as resulting from several factors, among which are personal health/behavioral practices such as smoking. In this study, the authors hypothesized that smoking, as a personal health/behavioral practice, is a factor in poor dental utilization and fewer dental visits for veterans based upon previous studies of other groups.

While there is a lack of similar studies among veterans, this study's results corroborate findings from research studies not specific to veterans. For example, researchers of a study of 15,250 U.S. adults, using MEPS 2000 data, reported that current smokers were less likely to have had a dental visit within the previous year than nonsmokers [AOR 0.78; 95% CI: 0.69, 0.88] [29]. Our study's results also support the findings from another study of 2,119 U.S. adults that showed that long-term smokers were less likely to have had dental care utilization in the previous year than never smokers [AOR: 0.69; 95% CI: 0.48, 0.99] [12].

Bloom et al. [30] reported that current smokers were more likely to delay dental visits. In a study using Behavioral Risk Factor Surveillance System (BRFSS) trend data (1995–2008), never smokers reported a higher median annual dental visit as compared with former smokers (3% difference) [31]. Current smokers were also more likely to visit only in case of dental emergency [9].

However, some researchers reported results dissimilar to this study. In a Japanese study, current smokers had more dental visits within the previous year as compared with nonsmokers (*P*=0.0003) [32]. Using 2014 BRFSS data, researchers found that smokers who were attempting to quit were more likely to have a recent dental visit [33].

More research is needed to understand the smoking-dental care utilization relationship. Statistically significant reasons for low dental care utilization include dental anxiety and financial barriers [34]. Specifically for U.S. veterans, dental coverage eligibility requirements are stringent [35]. Any needed dental care is provided to service-connected dental disability, to disabilities which are 100% disabling, or to veterans who were former prisoners of war [35]. However, exclusions apply for veterans who do not meet those criteria [35]. The implication is that access to needed dental care is limited.

The Office of the Actuary, the Department of Veterans Affairs has projected a steeper growth in the female, minority, and younger age veterans between 2010 and 2040 [36]. Given a new profile in the coming decades, researchers will need to reassess veterans' oral health needs. The Department of Veterans Affairs policy makers may want to incorporate features of successful dental service models across the country that proactively supports Veterans [37].

Dentists and dental healthcare professionals are in the position and have the ability to help with tobacco cessation [38] as many people have routine dental utilization. However, access to this specific population is problematic due to the lower dental utilization. Although not a focus of this study, higher education increased the likelihood of veterans attending dental visits and higher income increased the likelihood of veterans attending dental visits. Therefore, it is particularly important to target dental public health messages concerning the importance of dental visits to the groups who would most benefit from that knowledge and encourage them to have routine dental care.

### 4.1. Strengths and Limitations

This study has several strengths. Researchers used a large, nationally representative sample of U.S. veterans in which weights were applied to maintain population estimates in the data analyses. The survey questions made it possible to determine participants who were current smokers, former smokers, and who never smoked. The authors were able to indicate the independent effect of smoking, while controlling for other potentially confounding variables. The results that veterans who smoke are less likely to have dental care utilization within the previous six months may be helpful to dental care providers and policy makers to create focused interventions. The veteran health is a major concern and access to quality care is challenging. There have been many changes since the NSV2010 was completed. The NSV2010 was the most current of the NSV surveys to use. There is a need for continued surveillance in this important group.

As a cross-sectional study, the results cannot be interpreted as causal. In addition, the data on both key variables, dental care utilization and smoking status, were self-reported. Since smoking is socially unacceptable, participants may have underreported their smoking status. The dental care utilization data included an aggregation of all dental services. Since the researchers could not distinguish among the services, the assessment of the frequency of specific dental visits could not be determined. The dental utilization question also had a 6-month time frame imposed upon it. Health self-perception was used as specific co-morbidities that were not available in the original dataset; however, in sensitivity analysis excluding overall health, the results remained similar to those reported. Additionally, other covariates (metropolitan versus rural status, number of missing teeth) would have strengthened the study but also were not available. However, in the primary dataset, the purpose was not to capture co-morbidities, so questions about hypertension, diabetes, cardiovascular disease, obesity, etc. were not posed to the participants.

## 5. Conclusion

Based upon the Andersen Theoretical Model, in this study of 6,308 veterans, those who smoked were more likely to *not* have dental care utilization within the previous six months, indicating that personal health/behavioral factors influence not only general health but also oral health. These findings have implications for oral conditions. Since smokers are less likely to have routine dental visits, they are at higher risk for late diagnosis of their dental problems or other oral conditions such as potentially malignant and malignant lesions [39]. Veterans who smoke should be specifically targeted with promotional messages regarding oral healthcare utilization.

## Figures and Tables

**Table 1:** Sample characteristics of veterans from the National Survey of Veterans, 2010.

All	Total	
	N = 6,261	wt.% = 100.0
*Sex*		
Female	412	8.4
Male	5,849	91.6
*Race/ethnicity*		
White	5,719	86.9
Other race	542	13.1
*Age in years*		
50 and above	2,862	39.2
35 to less than 50	2,322	33.8
20 to less than 35	1,077	27.0
*Marital status*		
Married	4,650	71.1
No	1,611	28.9
*Education*		
Less than HS/HS degree	1,938	30.9
Some college	1,847	30.8
College degree/above	2,476	38.2
*Income*		
Less than $20,000	769	13.8
$20,000 to less than $30,000	814	12.8
$30,000 and above	4,678	73.4
*Insurance*		
Adequate	4,203	66.2
Inadequate	2,058	33.8
*Health self-perception*		
Excellent/very good/good	4,499	73.0
Fair/poor	1,762	27.0
*Smoking status*		
Current smoker	1,099	19.9
Past smoker	3,025	44.6
Never smoked	2,137	35.5
*Oral health perception*		
Excellent/very good/good	3,675	59.1
Fair/poor	2,586	40.9
*Mailing address*		
Rural route	4,737	80.1
Street address	671	6.5
U.S. PO box/other box	29	0.3
Missing	814	13.2
*Dental visit in previous 6 months*		
Yes	3,684	55.3
No	2,577	43.7

*Note.* Based on 6,261 eligible veterans. Abbreviations: wt, weighted; HS, high school.

**Table 2:** Veteran reports of dental care utilization within the previous 6 months by the National Survey of Veterans, 2010.

All	Not reported dental care utilization within 6 months		Reported dental care utilization within 6 months		*P* value
	*N*	wt.%	*N*	wt.%	
*Sex*					0.8104
Male	2,414	43.8	3,435	56.0	
Female	163	43.0	249	57.0	
*Race/ethnicity*					<0.0001
White	2,277	41.5	3,442	58.5	
Other	300	58.5	242	41.5	
*Age in years*					<0.0001
50 and above	1,127	40.1	1,735	59.9	
35 to less than 50	924	41.5	1,398	58.5	
20 to less than 35	526	51.6	551	48.4	
*Marital status*					<0.0001
Married	1,715	38.7	2,935	61.3	
No	862	56.1	749	43.9	
*Education*					<0.0001
Less than HS/HS degree	1,109	59.7	829	40.3	
Some college	817	46.5	1,030	53.5	
College degree/above	651	28.5	1,825	71.5	
*Income*					<0.0001
Less than $20,000	542	73.6	227	26.4	
$20,000 to less than $30,000	474	59.4	340	40.6	
$30,000 and above	1,561	35.3	3,117	64.6	
*Insurance*					<0.0001
Adequate	1,447	36.6	2,756	63.4	
Inadequate	1,130	57.6	928	42.4	
*Health self-perception*					<0.0001
Excellent/very good/good	1,619	39.2	2,880	60.8	
Fair/poor	958	55.9	804	44.1	
*Smoking status*					<0.0001
Current smoker	658	63.0	441	37.0	
Past smoker	1,162	39.9	1,863	60.1	
Never smoked	757	37.7	1,380	62.3	
*Oral health perception*					<0.0001
Excellent/very good/good	1,099	33.1	2,576	66.9	
Fair/poor	1,478	59.1	1,108	40.9	
*Mailing address*					0.0002
Rural route	1,906	42.7	2,841	57.3	
Street address	264	42.3	407	57.7	
PO box	^*∗*^		^*∗*^		
Missing	399	50.7	417	49.3	

*Note.* Based on 6,261 eligible veterans. Abbreviations: wt.%, weighted percent; HS, high school. *P* value is based upon Rho-Scott chi-square. ^*∗*^Cell sizes suppressed due to small cell sizes.

**Table 3:** Logistic regression of smoking on no reports of dental utilization within the previous 6 months (the National Survey of Veterans, 2010).

	Unadjusted		Adjusted	
	OR [95% CI]	*P* value	OR [95% CI]	*P* value
*Smoking status*				
*Current smoker*	2.81 [2.34, 3.38]	<0.0001	1.50 [1.22, 1.84]	<0.0001
*Past smoker*	1.09 [0.96, 1.26]	0.1874	0.99 [0.85, 1.15]	0.8963
*Never smoked*	Reference		Reference	
*Sex*				
Male			1.21 [0.90, 1.62]	0.2045
Female			Reference	
*Race/ethnicity*				
White			Reference	
Other			1.37 [1.08, 1.74]	0.0105
*Age in years*				
50 and above			0.59 [0.48, 0.72]	<0.0001
35 to less than 50			0.60 [0.50, 0.74]	<0.0001
20 to less than 35			Reference	
*Marital status*				
Married			Reference	
No			1.24 [1.06, 1.47]	0.0073
*Education*				
Less than HS/HS degree			2.41 [2.05, 2.83]	<0.0001
Some college			1.53 [1.30, 1.81]	<0.0001
College degree/above			Reference	
*Income*				
Less than $20,000			2.69 [2.14, 3.38]	<0.0001
$20,000 to less than $30,000			1.88 [1.54, 2.29]	<0.0001
$30,000 and above			Reference	
*Insurance*				
Adequate			Reference	
Inadequate			1.19 [1.03, 1.38]	0.0215
*Health self-perception*				
Excellent/very good/good			Reference	
Fair/poor			1.02 [0.87, 1.19]	0.8104
*Oral health perception*				
Excellent/very good/good			Reference	
Fair/poor			2.04 [1.77, 2.36]	<0.0001

*Note.* Based on 6,261 eligible veterans. Abbreviation: HS, high school.

## Data Availability

Previously reported 2010 National Survey of Veterans data were used to support this study and are available at https://catalog.data.gov/dataset/national-survey-of-veterans-baea8. This dataset is cited at relevant places within the text as Reference [28].

## Additional Points

*Practical Implications*. Population subgroups often have factors which influence healthcare more so than in the population at large. The association of smoking and dental visits is such an example in the veteran subgroup.
